# Curcumin Attenuates Gentamicin-Induced Kidney Mitochondrial Alterations: Possible Role of a Mitochondrial Biogenesis Mechanism

**DOI:** 10.1155/2015/917435

**Published:** 2015-08-05

**Authors:** Mario Negrette-Guzmán, Wylly Ramsés García-Niño, Edilia Tapia, Cecilia Zazueta, Sara Huerta-Yepez, Juan Carlos León-Contreras, Rogelio Hernández-Pando, Omar Emiliano Aparicio-Trejo, Magdalena Madero, José Pedraza-Chaverri

**Affiliations:** ^1^Departamento de Biología, Facultad de Química, UNAM, 04510 Ciudad de México, DF, Mexico; ^2^Departamento de Biomedicina Cardiovascular, Instituto Nacional de Cardiología “Ignacio Chávez”, 14080 Ciudad de México, DF, Mexico; ^3^Laboratorio de Fisiopatología Renal, Instituto Nacional de Cardiología “Ignacio Chávez”, 14080 Ciudad de México, DF, Mexico; ^4^Unidad de Investigación en Enfermedades Oncológicas, Hospital Infantil de México “Federico Gómez”, 06720 Ciudad de México, DF, Mexico; ^5^Departamento de Patología, Instituto Nacional de Ciencias Médicas y Nutrición “Salvador Zubirán”, 14000 Ciudad de México, DF, Mexico; ^6^Departamento de Nefrología, Instituto Nacional de Cardiología “Ignacio Chávez”, 14080 Ciudad de México, DF, Mexico

## Abstract

It has been shown that curcumin (CUR), a polyphenol derived from *Curcuma longa*, exerts a protective effect against gentamicin- (GM-) induced nephrotoxicity in rats, associated with a preservation of the antioxidant status. Although mitochondrial dysfunction is a hallmark in the GM-induced renal injury, the role of CUR in mitochondrial protection has not been studied. In this work, LLC-PK1 cells were preincubated 24 h with CUR and then coincubated 48 h with CUR and 8 mM GM. Treatment with CUR attenuated GM-induced drop in cell viability and led to an increase in nuclear factor (erythroid-2)-related factor 2 (Nrf2) nuclear accumulation and peroxisome proliferator-activated receptor gamma coactivator-1 alpha (PGC-1*α*) cell expression attenuating GM-induced losses in these proteins. *In vivo*, Wistar rats were injected subcutaneously with GM (75 mg/Kg/12 h) during 7 days to develop kidney mitochondrial alterations. CUR (400 mg/Kg/day) was administered orally 5 days before and during the GM exposure. The GM-induced mitochondrial alterations in ultrastructure and bioenergetics as well as decrease in activities of respiratory complexes I and IV and induction of calcium-dependent permeability transition were mostly attenuated by CUR. Protection of CUR against GM-induced nephrotoxicity could be in part mediated by maintenance of mitochondrial functions and biogenesis with some participation of the nuclear factor Nrf2.

## 1. Introduction

Curcumin (CUR) is the most active compound in* Curcuma longa* (turmeric or curcuma), an herbaceous plant popularly used as a culinary spice and traditional remedy. Chemically, curcumin is a bis-*α*, *β*-unsaturated *β*-diketone that also features two methoxy groups, two phenolic hydroxyl groups, and two double-conjugated bonds that play important roles in its well-known physiological benefits [1]. Among its reported biological properties, curcumin includes anticarcinogenic [2, 3], anti-inflammatory [4], antimicrobial [5], antiatherosclerotic [6], and antifibrotic [7] effects. Most of the anti-inflammatory and cytoprotective reports have been linked to the antioxidant activity of curcumin, which is a bifunctional antioxidant [8]. On one hand, curcumin can react directly with reactive oxygen species (ROS) thanks to the phenolic groups in its structure. On the other hand, curcumin is able to induce an upregulation of various cytoprotective and antioxidant proteins by activation of nuclear factor (erythroid-2)-related factor 2 (Nrf2), a master regulator of the cell antioxidant response [1, 9].

As was documented by our group and others, curcumin protective properties against different kinds of damage models in several tissues might be also mediated by mechanisms that involve preservation in mitochondrial integrity and functions [10]. Indeed, fails in heart performance induced by cardiac reperfusion [11] and 5/6-nephrectomy in rats [12] were improved with curcumin treatment in correlation with attenuated oxidative stress, recovery of antioxidant enzymes activities, and preservation of the respiratory capacity in isolated mitochondria. In addition, cardiotoxicity induced by catecholamine [13] and anoxia-reoxygenation in rats [14] was attenuated by curcumin-mediated inhibition of the mitochondrial permeability transition (MPT) pore and preserving energy production, respectively. In rat kidney and liver, hexavalent chromium-induced injury was attenuated through maintenance of bioenergetic status, calcium retention capacity, and activity of the respiratory complexes [15, 16]. Pretreatment with curcumin rendered similar results in a rat model of indomethacin-induced enteropathy [17]. Interestingly, Kuo et al. [18] and Liu et al. [19] found that the curcumin treatment normalized the mitochondrial biogenesis altered in a liver steatosis obese mice model and a cerebral ischemia reperfusion rat model, respectively, as markers like nuclear respiratory factor 1 (NRF1) and mitochondrial transcription factor A (Tfam) were preserved after the treatment.

Renal protection by curcumin has been well established [1]. There are numerous animal trials that showed nephroprotection by curcumin in some common health complications and in exposures to drugs and chemicals. For its clinical relevance, the protective effect of curcumin against gentamicin- (GM-) induced renal injury is highlighted [20, 21]. Nephrotoxicity is one of the main side effects of this aminoglycoside antibiotic and occurs in 10%–20% of therapeutic regimes. GM is one of the best known nephrotoxic drugs and its association with mitochondrial dysfunction in proximal tubules has been well studied [22]. Mitochondrial membrane potential disruption [23], oxygen consumption anomalies, adenosine triphosphate (ATP) yield decrease, MPT pore formation, mitochondrial cytochrome c release, intrinsic apoptosis, and mitochondrial antioxidant status impairment have been observed both in culture tubular cells and in rat kidneys exposed to GM [23–26]. On the other hand, some reports suggest that curcumin protective effect against GM nephrotoxicity is associated with preservation of the renal antioxidant status and with modulation of the inflammatory response mediated by NF-*κ*B, rather than with protection of renal mitochondrial function [20, 21, 27].

In the present work, we investigate the unexplored effect of curcumin treatment on alterations induced by GM in renal mitochondria in both cells and rats. Because inducers of nuclear translocation of Nrf2 like curcumin have been proposed as inducers of mitochondrial biogenesis [28], we evaluated Nrf2 and peroxisome proliferator-activated receptor gamma coactivator-1 alpha (PGC-1*α*) expression. PGC-1*α* is a central stimulator of mitochondrial biogenesis [29]. Ultrastructural mitochondrial changes, bioenergetic status, and respiratory complexes activities as well as calcium-dependent MPT pore opening were also evaluated. Our results evidence that the protective effect of CUR against GM-induced renal injury and dysfunction could be mediated by maintenance of kidney mitochondrial biogenesis, structure, and functions.

## 2. Materials and Methods

### 2.1. Chemicals and Reagents

CUR (Cat. no. C1386, batch 081M1611V), 3-(4,5-dimethylthiazol-2-yl)-2,5-diphenyltetrazolium bromide (MTT), dimethyl sulfoxide (DMSO), glutaraldehyde, sodium cacodylate, osmium tetroxide (OsO_4_), paraformaldehyde, bovine serum albumin, potassium succinate, sodium glutamate, sodium malate, adenosine diphosphate (ADP), N-(2-hydroxyethyl)piperazine-N′-(2-ethanesulfonic acid) (HEPES), rotenone, carbonyl cyanide m-chlorophenylhydrazone (CCCP), decylubiquinone, nicotinamide adenine dinucleotide reduced form (NADH), potassium cyanide (KCN), antimycin A, sucrose, phenazine methosulfate (PMS), cytochrome c from equine heart, ascorbic acid, tetramethyl-p-phenylenediamine (TMPD), manganese(II) chloride tetrahydrate, safranin O, arsenazo III, cyclosporine A (CsA), 3-(N-morpholino) propane-sulfonic acid (MOPS), and ethylene glycol-bis(2-aminoethylether)-*N,N,N*′*,N*′-tetraacetic acid (EGTA) were purchased from Sigma-Aldrich (St. Louis, MO, USA). GM (Garamicina G.U. 120 mg/1.5 mL, batches 2DPDA006 and 1DPDA002) was obtained from Schering-Plough (Mexico City, DF, Mexico). Dulbecco's Modified Eagle Medium (DMEM), fetal bovine serum (FBS), trypsin, antibiotic (10,000 U/mL penicillin and 10,000 *μ*g/mL streptomycin), and other tissue culture reagents were purchased from Gibco (Mexico City). Rabbit polyclonal anti-Nrf2 antibody (C-20, Cat. no. sc-722) was obtained from Santa Cruz Biotechnology, Inc. (Santa Cruz, CA, USA). Rabbit polyclonal anti-PGC-1*α* antibody (Cat. no. ab54481) was purchased from Abcam, Inc. (Cambridge, MA, USA). Normal goat serum blocking solution (S-1000) and Avidin/Biotin Blocking Kit (SP-2001) were obtained from Vector Laboratories, Inc. (Burlingame, CA, USA). Biotinylated Link Universal, Streptavidin-HRP, and 3,3′-diaminobenzidine (DAB) were obtained from Dako (Carpinteria, CA, USA). Potassium chloride (KCl), sodium citrate, dextrose, and ethylenediaminetetraacetic acid disodium salt dihydrate (EDTA) were acquired from J.T. Baker (Xalostoc, Edo. Mex, Mexico). All other reagents and chemicals used were of the highest grade of purity commercially available.

### 2.2. Cell Culture and Viability

Lily Laboratory Culture Porcine Kidney (LLC-PK1) porcine renal epithelial cells were obtained from American Type Culture Collection (Rockville, MD, USA). This cell line is an accepted model to study toxicity of aminoglycosides [23, 30]. LLC-PK1 cells were maintained in DMEM supplemented with 10% FBS and 1% of antibiotic and cultured under permissive conditions: 37°C and 5% CO_2_. In order to evaluate the effect of CUR on GM-induced toxicity, cells were seeded at a density of 3 × 10^4^ cells/cm^2^ onto either 96-well or 6-well plates and used for the experiment on the following day. Cells were incubated for 24 h with CUR (10–30 *μ*M) or medium before the GM addition. At the end of preincubation period, CUR or culture medium was replaced by fresh CUR or medium, adding 8 mM GM to some cell groups in order to induce toxicity. CUR treatment and GM exposure were maintained for 48 h by replacing the first medium with fresh medium at 24 h. Cell viability was assessed by MTT reduction. At the end of 72 h of experiment, medium was removed and cells were washed twice with phosphate-buffered saline (PBS) pH 7.4. In 96-well plates, cells were incubated in medium containing MTT (0.125 mg/mL) at 37°C for 1 h in humidified air supplemented with 5% CO_2_. Medium was then discarded and the formazan crystals deposited in each well bottom were dissolved in 100 *μ*L of 0.1 N HCl in isopropanol. Absorbance was determined at 570 nm using an EnSpire multimode plate reader (PerkinElmer Inc., Waltham, MA, USA). Cells incubated in 6-well plates were trypsinized and resuspended in PBS at a proportion of 20 000 cells/10 *μ*L. Spots of 20 000 cells were seeded on slides, left drying at room temperature, fixed in 4% formaldehyde at 4°C, and washed three times in PBS for posterior immunocytochemical analysis.

### 2.3. Immunocytochemistry

Immunocytochemical staining for Nrf2 and PGC-1*α* was performed in LLC-PK1 cells fixed with formaldehyde pH 7.4 on slides. Antigens were recuperated by boiling for 20 min in 0.01% sodium citrate solution, pH 6.0. Background staining was reduced by blocking with 3% H_2_O_2_ solution in methanol for 30 minutes, incubation in a 2% solution of normal goat serum in PBS (PBS-NGS) for 2 hours, and treatment with avidin and biotin for 10 min each. Slides were incubated overnight at room temperature with anti-Nrf2 (1 : 100) and anti-PGC-1*α* (1 : 250) primary antibodies. The following day, slides were washed five times for 5 min in PBS 1X pH 7.4. After washing, slides were incubated for 30 min at room temperature with universal biotinylated link and for 30 min at room temperature with streptavidin conjugated to HRP. For color developing, DAB was used from 1 to 5 min. The reaction was stopped with distilled water and the slides were counterstained with hematoxylin. Finally, cells were dehydrated and fixed with Mount E-2 medium (Shandon Laboratory, Pittsburgh, PA, USA). Slides were analyzed under a microscope Olympus BX40 and immunopositive cells were quantified by simple counting.

### 2.4. Animals

Male Wistar rats with an initial body weight of 200–220 g were used. Animals were maintained under 12-h light/dark cycles at controlled temperature, having* ad libitum* access to water and standard food. Local Committee for the Care and Use of Laboratory Animals approved this experimental study (FQ/CICUAL/038/12), which was conducted according to the guidelines of Mexican Official Norm Guide for the use and care of laboratory animals (NOM-062-ZOO-1999) and for the disposal of biological residues (NOM-087-SEMARNAT-SSA1-2002).

### 2.5. Experimental Design

Animals were randomly divided into four groups: (i) control group (CT) was injected subcutaneously (s.c.) with isotonic saline solution (ISS, vehicle for GM) every 12 h for 7 days and administered with carboxymethyl cellulose (vehicle of CUR) by oral gavage once a day during five days previous to any ISS injection and between the daily ISS injections. (ii) Gentamicin group (GM) was administered s.c. with GM at a dose of 75 mg/Kg/12 h [31] and carboxymethyl cellulose was given like in CT group. (iii) CUR + GM group was injected with GM as in the GM group but received oral CUR (400 mg/Kg) in carboxymethyl cellulose [15] 5 days before GM exposure and between the two daily GM injections (14 doses). (iv) CUR group was administered s.c. with ISS during 7 days and with CUR during 12 days. On the thirteenth day of treatment, rats were euthanized by anesthetization with sodium pentobarbital (60 mg/Kg) and bled via abdominal aorta using a syringe containing heparin and a needle #18 at room temperature. Plasma was separated and stored at −20°C until the markers of renal damage, plasma creatinine, and blood urea nitrogen (BUN) were measured.

### 2.6. Analytical Methods

Creatinine and BUN in plasma were determined by spectrophotometric assays using commercial Spinreact kits as previously reported [23]. Creatinine determination in plasma is based on the reaction of this compound with sodium picrate forming a red complex whose intensity is proportional to the creatinine concentration. However, urea present in the plasma reacts with o-phthalaldehyde forming a colored complex which is quantified at 510 nm.

### 2.7. Ultrastructural Study

To study the mitochondrial ultrastructural morphology, immediately after animal sacrifice, thin kidney tissue slices were obtained and immersed into 4% glutaraldehyde dissolved in 1 mM cacodylate buffer pH 7.2. Then, the kidney cortex was selected and sectioned in small tissue fragments that were deposited into glass tubes and fixed by immersion in the same solution during 24 hr at 4°C. Then, tissue fragments were postfixed with 2% OsO_4_ buffer, dehydrated in graded ethyl alcohol solutions, and embedded in Epon Resin (London Resin Company, London, UK). Thin sections from 70 to 90 nm were placed on cooper grids, contrasted with lead and uranium salts, and examined with a FEI Tecnai G2 Spirit transmission electron microscope (Hillsboro, OR, USA).

### 2.8. Mitochondria Isolation

Kidneys were quickly removed and maintained in cold isolation buffer (250 mM sucrose, 10 mM HEPES, 1 mM EGTA, pH 7.3). The renal cortex was separated and utilized for mitochondria isolation. Tissue was ground and then homogenized in a Glass/Teflon Potter Elvehjem homogenizer in the same buffer. Mitochondria were obtained by differential centrifugation and the protein content was measured by Biuret method [15].

### 2.9. Mitochondrial Bioenergetics

Oxygen consumption was measured using a Clark type oxygen electrode (Yellow Springs Instruments, Yellow Springs, OH, USA) and two different respiratory substrates. To evaluate respiration driven by complex I, 1 mg of mitochondrial protein was added to 1.7 mL of basic medium containing 125 mM KCl, 10 mM HEPES, 3 mM inorganic phosphate (Pi), 10 mM sodium malate, and 10 mM sodium glutamate at pH 7.3. Oxygen consumption sustained by complex II was evaluated replacing malate and glutamate with 10 mM succinate plus 1 *μ*g/mL rotenone. State 4 was registered in basic medium, while state 3 respiration was stimulated with ADP (final concentration 200 *μ*M) [32]. Respiratory control index (RCI) was calculated as the ratio state 3/state 4. Uncoupled respiration was obtained by adding 1 *μ*M of CCCP. Phosphorylation efficiency (ADP/O ratio) was calculated from the added amount of ADP and the total amount of oxygen utilized during the developed state 3 [16].

### 2.10. Activity of Mitochondrial Respiratory Complexes and Aconitase

Effects on mitochondrial enzyme activities were evaluated as previously described [15]. Complex I activity was measured by following the decrease in absorbance due to oxidation of NADH to NAD^+^ at 340 nm. The reaction was initiated by adding 60 *μ*M decylubiquinone in 1.7 mL of standard reaction medium (125 mM KCl, 10 mM HEPES, 3 mM inorganic phosphate, pH 7.3) supplemented with 0.1 *μ*g antimycin A, 1 mM KCN, 100 *μ*M NADH, and 0.5 mg of mitochondrial protein. Complex II activity was determined polarographically by recording oxygen consumption. PMS was used as an artificial electron acceptor and succinate as a donor. The reaction was initiated by adding 1 mM PMS in 1.7 mL of standard reaction medium supplemented with 5 mM succinate, 2 *μ*M rotenone, 0.1 *μ*g antimycin A, 1 mM KCN, 1 *μ*M CCCP, and 0.5 mg of mitochondrial protein. Complex III activity was performed by following the increase in absorbance at 550 nm resulting from the reduction of cytochrome c. The assay included oxidized cytochrome c as electron acceptor and decylubiquinol as donor. The reaction was carried out in 2 mL of reaction medium (25 mM K_2_HPO_4_, 1 mM EDTA, pH 7.6) supplemented with 1 mM KCN, 20 *μ*M cytochrome c, 2 *μ*M rotenone, and 10 *μ*g of mitochondrial protein. The reaction was initiated by the addition of 25 *μ*M decylubiquinol. An extinction coefficient value of 18.7 mM^−1^ cm^−1^ was used for reduced cytochrome c. Complex IV activity was followed polarographically. TMPD was used as an artificial electron mediator that accelerates the transfer of electrons from ascorbate to membrane-bound cytochrome c. The reaction was carried out in 1.7 mL of the standard reaction medium supplemented with 5 mM ascorbic acid, 2 *μ*M rotenone, 1 *μ*M CCCP, 0.5 *μ*g antimycin A, 25 *μ*M cytochrome c, and 2.5 mM TMPD. The reaction was initiated by the addition of 50 *μ*g of mitochondrial protein. The activity of aconitase was assayed by determining the rate of formation of the intermediate product,* cis*-aconitate, from the interconversion of L-citrate and isocitrate at 240 nm. Briefly, the reaction was carried out in 1 mL of reaction medium containing 25 mM KH_2_PO_4_ + 0.05% Tween, 1 mM sodium citrate, and 0.6 mM MnCl_2_. The reaction was initiated by the addition of 50 *μ*g of mitochondrial protein. An extinction coefficient for* cis*-aconitate of 3.6 mM^−1^ cm^−1^ was used.

### 2.11. Ca^2+^-Dependent MPT

Effects on the opening of MPT pore induced by Ca^2+^ overload were evaluated in a double-beam spectrophotometer UV-2401 Shimadzu (Kyoto, Japan), as previously described [15]. Ca^2+^ retention capacity was determined monitoring the absorbance changes at 625–675 nm of the dye arsenazo III (60 *μ*M) in 2.8 mL of medium containing 125 mM KCl, 10 mM HEPES, 3 mM Pi, 10 mM succinate, 1.8 *μ*g/mL rotenone, and 200 *μ*M ADP (pH 7.3). Mitochondria (2 mg of mitochondrial protein) were added and then challenged with 100 *μ*M CaCl_2_. In a similar way, Ca^2+^-induced membrane potential dissipation was evaluated spectrophotometrically at 525–575 nm using 10 *μ*M safranin O. Both assays were also performed in presence of 1 *μ*M CsA, a known inhibitor of the MPT pore. Depolarization of the membrane potential was induced by CCCP addition at the end of the assays [15].

### 2.12. Statistics

Results are expressed as mean ± SEM. Data were analyzed by one-way ANOVA followed by Bonferroni's multiple comparisons test using the software Prism 5.0 (GraphPad, San Diego, CA, USA). A *P* value less than 0.05 was considered statistically significant.

## 3. Results and Discussion

### 3.1. Viability of LLC-PK1 Cells

**Figure 1:**
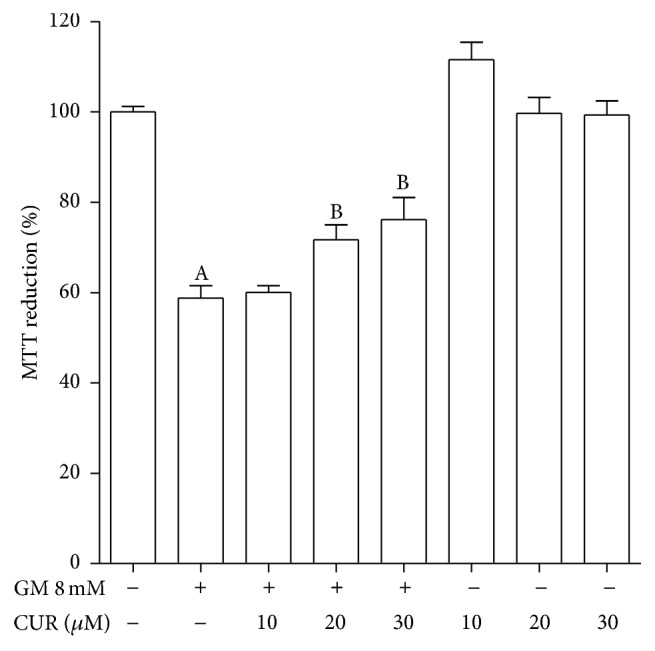
CUR attenuates GM-induced cell viability drop in LLC-PK1 cells. Cells were preincubated with 10–30 *μ*M CUR during 24 h and then coincubated with 10–30 *μ*M CUR and 8 mM GM during further 48 h. Viability was measured as percentage of MTT reduction compared to control cells (CT). Data are mean ± SEM, *n* = 3. ^A^ *P* < 0.001 versus CT; ^B^ *P* < 0.05 versus GM-treated cells.

Incubation for 48 h with GM decreased cell viability (expressed as MTT reduction) to 58.8% compared to untreated cells (Figure 1). A cytoprotective effect was observed when LLC-PK1 cells were pre- and coincubated with 20 and 30 *μ*M CUR (viabilities of 71.6% and 76.1%, resp.). Viability of cells only incubated with CUR was not significantly different from control cells. Minor concentrations were assayed but no protection was found. On the other hand, concentrations of 40 and 50 *μ*M were also tested but they were seemingly toxic as viability decreased in cells incubated only with CUR and the protection against GM was not observed (data not shown).

### 3.2. Nrf2 Nuclear Accumulation and PGC-1*α* Expression

**Figure 2:**
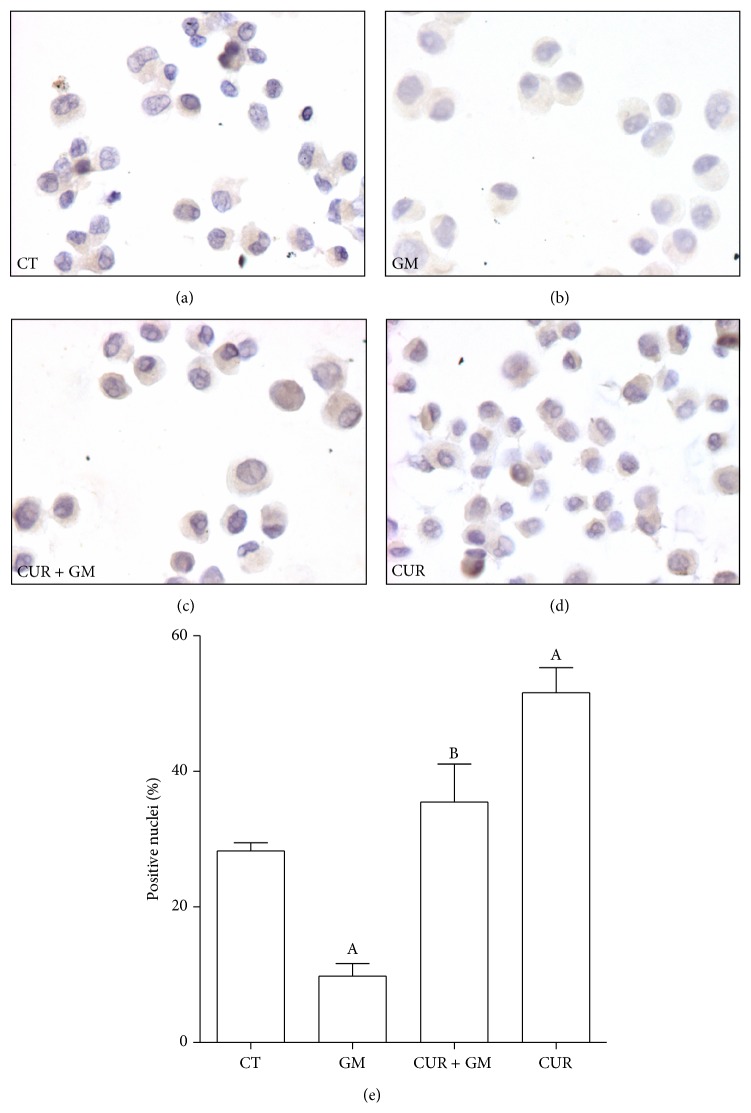
CUR induces an increase in nuclear accumulation of Nrf2 and prevented the GM-induced drop in Nrf2 expression and nuclear accumulation in LLC-PK1 cells. Cells were preincubated with 30 *μ*M CUR during 24 h and then coincubated with 30 *μ*M CUR and 8 mM GM during further 48 h. Expression of Nrf2 was detected by immunocytochemical technique. (a) CT. (b) GM. (c) CUR + GM. (d) CUR. (e) Quantification of positive nuclei. Data are mean ± SEM, *n* = 3. ^A^ *P* < 0.05 versus CT; ^B^ *P* < 0.01 versus GM.

CUR is a bifunctional antioxidant that induces nuclear accumulation of the factor Nrf2 in LLC-PK1 cells [33]. In order to confirm its role in this cell culture model, we evaluated the nuclear immunoprevalence of Nrf2 at the end of the CUR-GM scheme. After 72 h of 30 *μ*M CUR incubation, nuclear translocation of Nrf2 significantly increases compared to CT cells (Figures 2(a) and 2(d)). Nuclear accumulation is inhibited in GM cells as expression is mainly located in cytoplasm but not in nucleus (Figure 2(b)). Immunopositive nuclei fell from 28.3% in CT cells to 9.8% in GM cells (Figure 2(e)). However, pre- and coincubation with 30 *μ*M CUR in cells exposed to GM (CUR + GM cells) prevented this effect (*P* < 0.05). Hereafter, it could be considered that Nrf2 plays a role in the effects observed.

**Figure 3:**
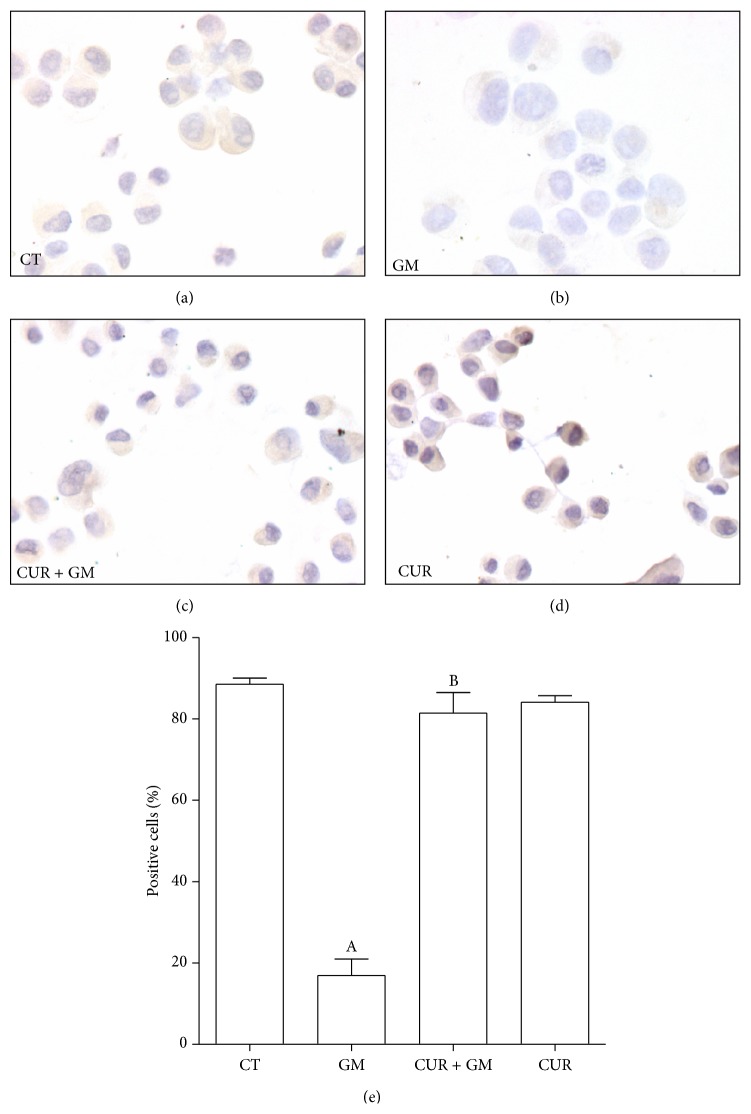
CUR prevents GM-induced drop in expression of the coactivator PGC-1*α* in LLC-PK1 cells. Cells were preincubated with 30 *μ*M CUR during 24 h and then coincubated with 30 *μ*M CUR and 8 mM GM during further 48 h. Expression of PGC-1*α* was detected by immunocytochemical technique. (a) CT. (b) GM. (c) CUR + GM. (d) CUR. (e) Quantification of positive cells. Data are mean ± SEM, *n* = 3. ^A^ *P* < 0.001 versus CT; ^B^ *P* < 0.001 versus GM.

The coactivator PGC-1*α* has been well identified as a potent inducer of mitochondrial biogenesis* trans*-activating target genes of nuclear factors like NRF1 and NRF2, involved in the program of respiratory gene expression. Also, PGC-1*α* induces transcripts of these factors, confirming its important integrative role upstream of biogenic program [29]. In Figures 3(a) and 3(b), it can be noticed that GM induced a strong drop in PGC-1*α*-expressing cells (from 88.5% in CT to 16.9% in GM). This effect was entirely prevented with the CUR treatment; nevertheless, it seems that CUR does not induce* per se* an increase in cells expressing PGC-1*α* (Figure 3(e)).

Connection between Nrf2 and mitochondrial biogenesis has been established. It was reported that 5′-UTR for NRF1 contains binding motifs for Nrf2, antioxidant response elements (AREs). Nuclear translocation of Nrf2 was followed by NRF1 induction and mitochondrial biogenesis that enabled rescuing mice from doxorubicin-induced cardiomyopathy and lethal* Staphylococcus aureus* sepsis, effects accompanied by an induction in PGC-1*α* [34, 35]. The conservation of PGC-1*α* levels in CUR + GM LLC-PK1 cells could be associated with the curcumin-induced Nrf2 nuclear translocation. This* in vitro* approach could work in involving mitochondrial biogenesis in the following observations* in vivo*.

### 3.3. Rat Renal Function

**Figure 4:**
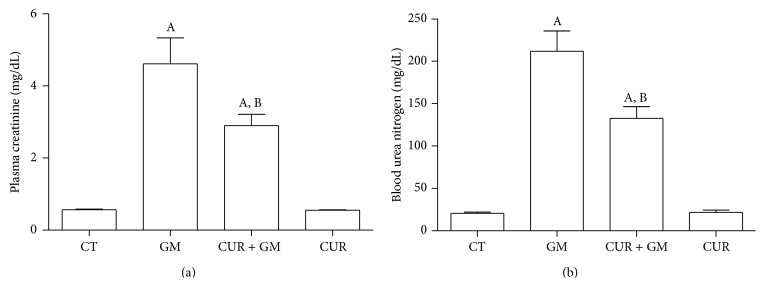
CUR ameliorates GM-induced renal dysfunction in rats. (a) Plasma creatinine. (b) Blood urea nitrogen. Data are mean ± SEM, *n* = 6. ^A^ *P* < 0.01 versus CT; ^B^ *P* < 0.05 versus GM.

Figure 4 shows the protective effect of CUR against GM-induced renal dysfunction in rats. GM induced an 8-fold increase in plasma creatinine level (Figure 4(a)). This notable GM-elicited change was attenuated in the CUR + GM group that showed plasma creatinine values 37% lower than those found in the GM group (*P* < 0.05 versus GM). A similar trend was observed in BUN (Figure 4(b)). An increase about 10-fold above CT value was obtained in the rats injected with GM, which was attenuated by 37% in the CUR + GM rats (*P* < 0.05). Animals administered only with CUR showed no changes in these parameters.

Previous works carried out by Ali et al. [20] and Farombi and Ekor [21] where a different GM administration scheme was used, with daily single doses of 80 mg/Kg intramuscularly during 6 days [20] and 100 mg/Kg intraperitoneally for 7 days [21], showed that creatinine and BUN levels in animals treated with GM increase 2–4.4-fold compared to CT group. Nevertheless, in rats cotreated with GM and CUR, those levels were similar to CT group. In our study, we observed that creatinine and BUN levels were about 10-fold greater in GM group than those observed in the CT animals after daily administration of 150 mg/Kg, which was distributed in two injections, to approach a clinical multidoses system. In this regard, some meta-analysis studies have shown that monodoses system (one daily single dose) correlates with less nephrotoxicity than multidoses system [36]. We choose the administration of a higher GM dose by a multidose system to achieve significant mitochondrial dysfunction and then evaluate the protective effect of curcumin in this condition. Successfully, CUR treatment ameliorates the GM-induced increase on both plasma creatinine and BUN levels in the GM + CUR group in spite of the high toxicity induced for this antibiotic.

### 3.4. Mitochondrial Ultrastructure

**Figure 5:**
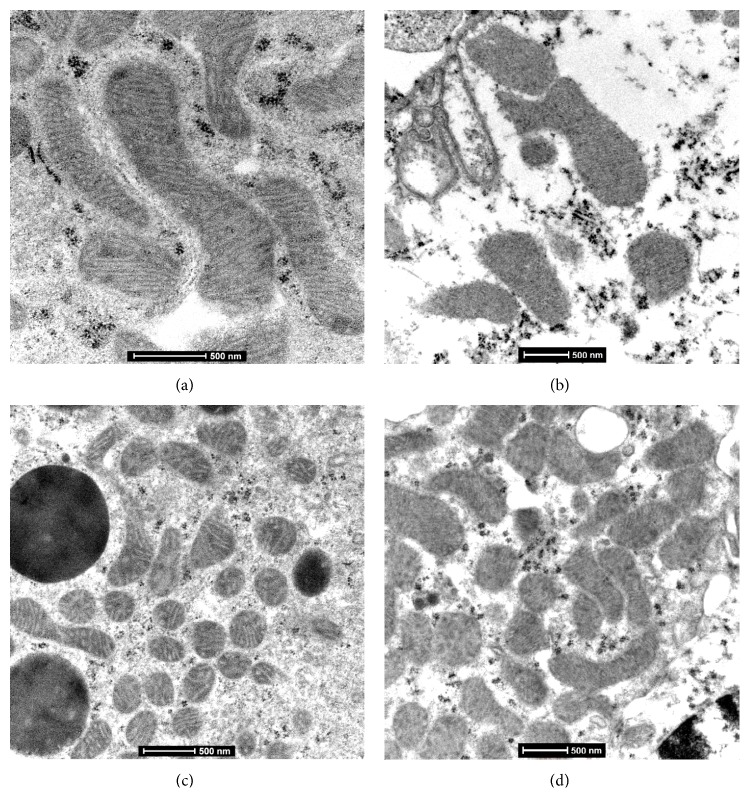
Representative micrographs of mitochondrial ultrastructure from convoluted proximal tubules. (a) Normal mitochondria structure in epithelial cell from convoluted proximal tubule from control rat. (b) In contrast, mitochondria show total cristae effacement in an animal treated with GM. (c) Convoluted proximal epithelial cell from a rat treated with CUR + GM shows numerous mitochondria with well-preserved morphology. (d) Similar mitochondrial morphology to CT animal is observed in rat only treated with CUR.

In well agreement with these determinations, the electron microscopy study showed extensive damage in the mitochondrial morphology produced by GM, characterized by effacement of inner membrane (cristae) (Figure 5(b)), while GM + CUR group showed higher number of mitochondria with almost normal structure; just some swollen mitochondria were seen (Figure 5(c)). The mitochondrial ultrastructure of proximal tubules from CT rats (Figure 5(a)) and treated with CUR (Figure 5(d)) was well preserved. In addition, it was observed that CUR treatment increases the number of rounded mitochondria having electron dense matrices and tightly packed cristae (Figures 5(c) and 5(d)).

In this connection, there are several* in vivo* studies on rodents that demonstrate the protective effect of CUR on mitochondrial ultrastructure injury [37–41], in a similar way to our results. Besides, it has been previously confirmed that CUR increases mitochondrial biogenesis [18, 42, 43]. Liu et al. [19] suggested that this mechanism could be responsible for neuroprotection in a model of ischemia/reperfusion on brain. Thus, CUR attenuates GM-induced mitochondrial alterations by a mitochondrial biogenesis mechanism.

### 3.5. Mitochondrial Bioenergetics

**Figure 6:**
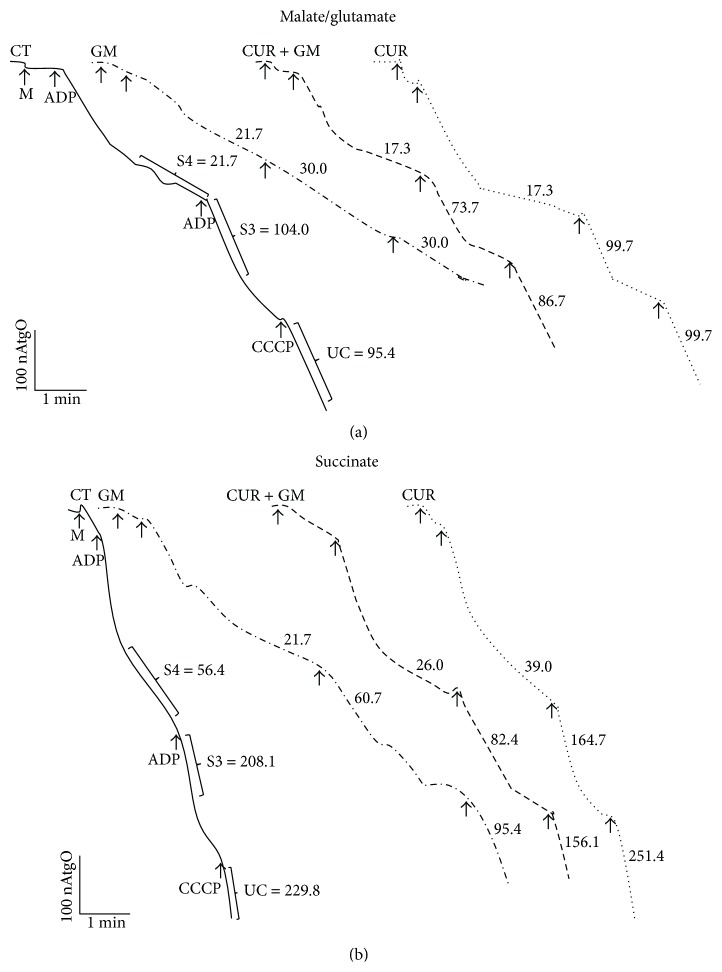
Representative tracings for oxygen consumption profiles of renal mitochondria using (a) malate/glutamate or (b) succinate as substrates. Values on tracings belong to punctual observations and are not means. M: mitochondria; ADP: adenosine diphosphate; CCCP: carbonyl cyanide m-chlorophenylhydrazone; S3: state 3; S4: state 4; UC: uncoupled respiration; CT: control; GM: gentamicin; CUR: curcumin. Units of S3, S4, and UC are ngAtO/min/mg protein.

Oxygen consumption profiles of rat renal mitochondria representatives of each experimental group are indicated for the two respiratory substrate conditions, malate/glutamate (Figure 6(a)) and succinate (Figure 6(b)). Oxygen consumption coupled to phosphorylation was reduced in mitochondria obtained from the GM-treated group as compared with CT mitochondria. CCCP stimulated respiratory rates in all conditions except in GM mitochondria oxidizing NADH-linked substrates (Figure 6(a)). At both substrates conditions, it can be graphically noted that the healthy behavior is partially preserved in mitochondria from rats cotreated with CUR and GM. Besides, there were no relevant differences between CUR and CT mitochondria (data not shown).

**Figure 7:**
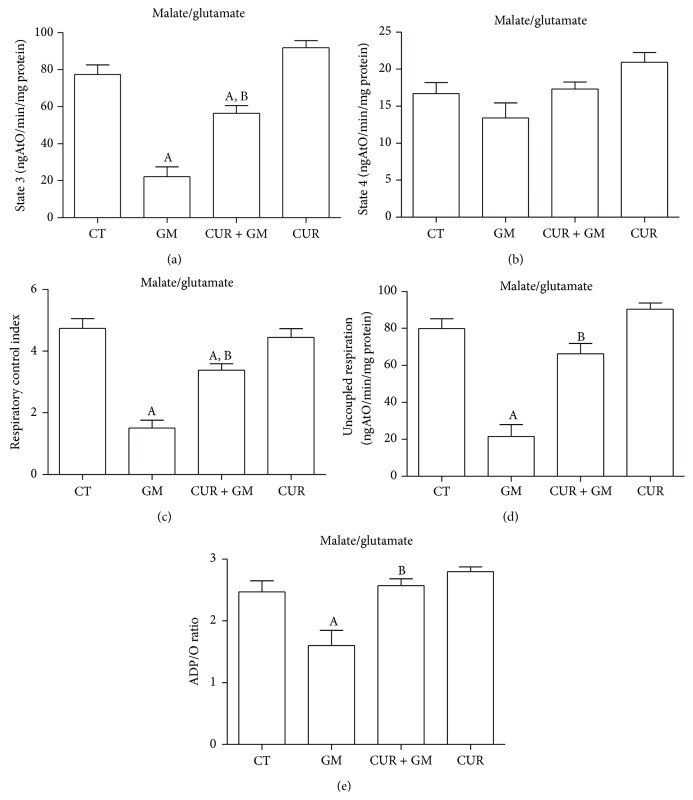
CUR attenuates GM-induced alterations in renal mitochondrial site I (malate/glutamate as a respiratory substrate) bioenergetics. (a) State 3. (b) State 4. (c) Respiratory control index. (d) Uncoupled respiration. (e) ADP/O ratio. Data are mean ± SEM, *n* = 6–8. ^A^ *P* < 0.05 versus CT; ^B^ *P* < 0.01 versus GM.

**Figure 8:**
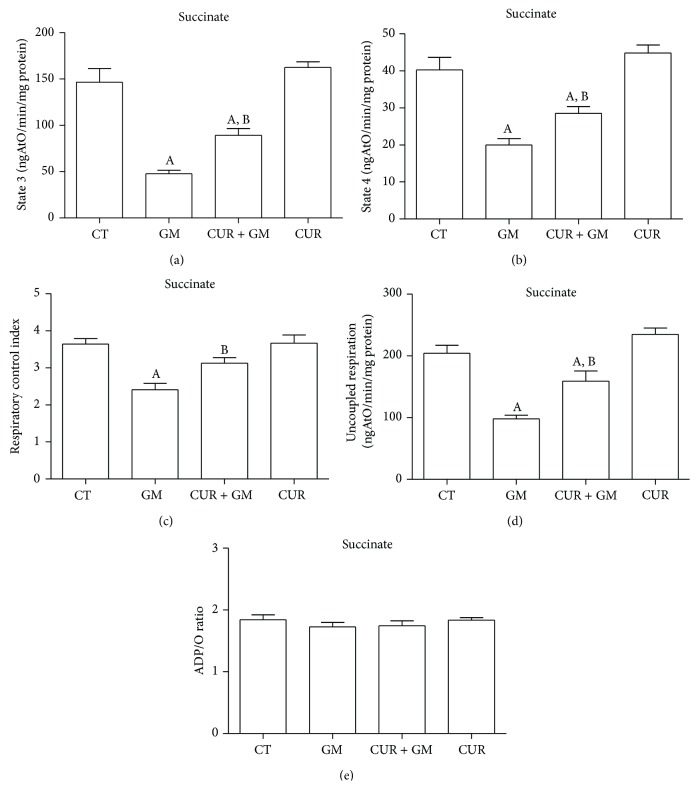
CUR attenuates GM-induced alterations in renal mitochondrial site II (succinate as a respiratory substrate) bioenergetics. (a) State 3. (b) State 4. (c) Respiratory control index. (d) Uncoupled respiration. (e) ADP/O ratio. Data are mean ± SEM, *n* = 6–8. ^A^ *P* < 0.05 versus CT; ^B^ *P* < 0.05 versus GM.

Outcomes for the major bioenergetics parameters obtained by oxygen consumption measurements are shown in Figure 7 (using malate/glutamate as substrate) and in Figure 8 (using succinate as substrate). State 3 decreased by 30% in mitochondria from rats treated with GM (Figures 7(a) and 8(a), *P* < 0.05 versus CT). ADP-stimulated oxygen consumption was preserved in CUR + GM by 73% in mitochondria supplied with malate/glutamate and around 61% in mitochondria supplied with succinate. Basal respiration was maintained in mitochondria from all groups fed with malate/glutamate (Figure 7(b)). In contrast, state 4 was lower in mitochondria from the GM group oxidizing succinate. State 4 rates were not completely reestablished in CUR + GM, while similar values of oxygen consumption between CT and CUR were observed under these conditions (Figure 8(b)). However, using either substrate, these values lead to significant differences in the RCI between the GM group and the CT group and between CUR + GM group and GM group. RCI falls from 4.7 in CT mitochondria to 1.4 (30%) in GM mitochondria and was recovered to 3.8 (81% compared with CT) in CUR + GM mitochondria (Figure 7(c)) with malate/glutamate, whereas RCI of GM group falls to 67% of CT and renal mitochondria from the CUR + GM group reached 86% of untreated rats RCI (Figure 8(c), *P* < 0.05 versus GM) when using succinate.

Uncoupled respiration was lower (24% of CT group) in GM mitochondria oxidizing malate/glutamate (Figure 7(d), *P* < 0.05 versus CT) and such diminution was prevented with curcumin. A similar trend was observed when succinate was supplemented, but the drop was lesser and was slightly attenuated by CUR treatment (Figure 8(d)).

ADP/O ratio in GM mitochondria oxidizing NADH-linked substrates showed lower phosphorylation rates as compared with CT mitochondria. Mitochondria from CUR + GM group recovered phosphorylation rates (Figure 7(e)). On the other hand, ADP/O values were similar in all experimental groups when succinate was the energizing substrate (Figure 8(e)). The CUR group had a behavior similar to that for CT group in all of evaluated bioenergetics parameters.

Oxidative phosphorylation is controlled by the activity of ATP turnover (adenine nucleotide translocase, phosphate transporter, and ATP synthase) and substrate oxidation (substrate uptake, processing enzymes, relevant electron-transport chain complexes, pool sizes of ubiquinone and cytochrome c, and O_2_ concentration) [44]. Simmons et al. [45] and Weinberg and Humes [46] determined that GM inhibits oxidative phosphorylation in renal cortical mitochondria related with the inhibition in maximal rates of electron transport mainly in the input components of the chain. Consequently, our results showed that mitochondria from GM-treated rats presented alterations in oxygen consumption by decreasing state 3 rates using malate/glutamate and succinate as substrates, which was noticeably recovered after curcumin treatment. Previously, it has been reported that curcumin restored mitochondrial state 3 rates in a model of renal dysfunction induced by hexavalent chromium [15]. On the other hand, in GM mitochondria, no significant change was registered on state 4 in glutamate/malate-dependent oxygen consumption. In contrast, state 4 in mitochondria from GM-treated rats presented lower values of oxygen consumption using succinate as a substrate. Morales et al. [25] reported a similar reduction on state 4 rates in rats treated with GM. Studies in isolated mitochondria from rat renal cortex incubated with GM showed increment in state 4 dependent on the antibiotic concentration [46, 47].

RCI is the single most useful general measure of function in isolated mitochondria. High RCI indicates good function and low RCI usually indicates uncoupling [44]. Mitochondria from GM-treated rats showed dysfunction when using both malate/glutamate or succinate and curcumin attenuated strongly such dysfunction. Uncoupled respiration was substantially reduced below CT values in GM group and recovered in CUR + GM group, mainly when malate/glutamate were used. This is a parameter controlled exclusively by substrate oxidation and detects dysfunction in respiratory chain components, substrate translocases, or dehydrogenases [44]. Accordingly, ADP/O ratio in mitochondria oxidizing malate/glutamate was decreased in GM group and recovered in mitochondria from cotreated rats. Curcumin prevents mitochondrial dysfunction by maintaining redox homeostasis or by protecting the mitochondrial respiratory complexes [17, 48]; nevertheless, biogenic recuperation of complexes could be another way.

### 3.6. Activity of Respiratory Complexes and Aconitase

**Figure 9:**
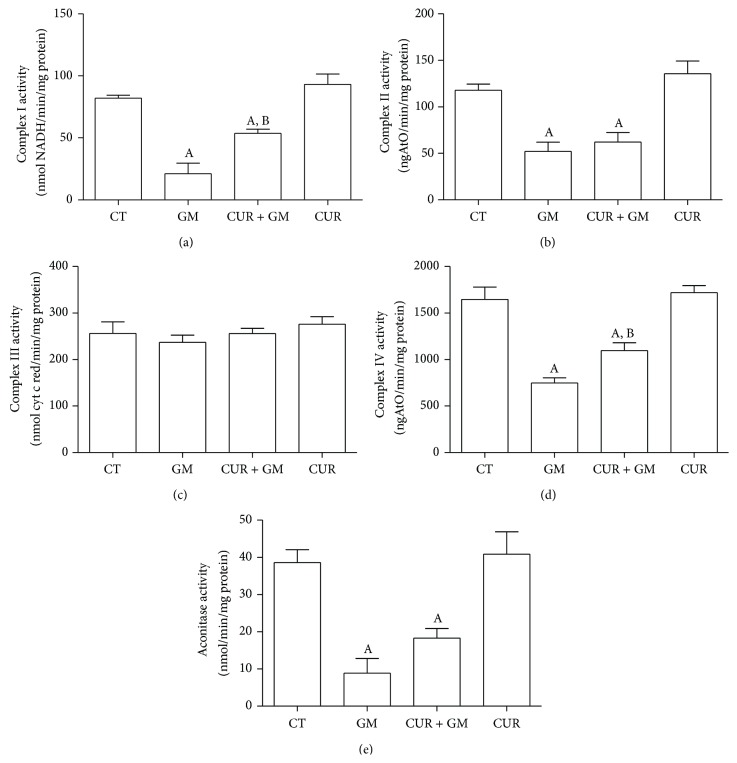
Effect of CUR treatment over GM-induced changes in the activity of renal respiratory complexes and of aconitase. (a) Complex I. (b) Complex II. (c) Complex III. (d) Complex IV. (e) Aconitase. Data are mean ± SEM, *n* = 5-6. ^A^ *P* < 0.05 versus CT; ^B^ *P* < 0.05 versus GM.

We also evaluated the activity of the respiratory complexes and found that GM induced reduction in the activity of complexes I, II, and IV. Activity of complex I in the GM group was the most affected retaining 25% of the activity observed in CT mitochondria (*P* < 0.05) (Figure 9(a)). Activity of complexes II and IV declined close to 45% of that observed in the CT group (Figures 9(b) and 9(d)). Curcumin treatment recovered the activities of complexes I and IV (Figures 9(a) and 9(d)), but no recovery was observed in complex II (Figure 9(b)). Retrievals reached approximately 67% of the respective CT value (*P* < 0.05 versus GM). Respiratory complexes from CUR-administered rats had similar activities to those corresponding to untreated animals.

In Figure 9(e) it is shown that the GM exposure in rats leads to a significant loss of aconitase activity in relation to the CT (*P* < 0.05). Even though a weak trend toward activity recuperation is observed in the CUR + GM group, this was not significantly different. Aconitase activity in mitochondria of rats treated only with CUR showed similar values to CT group.

We have already discussed the components of RCI, state 3 and state 4, and with the results of complexes activity assays we will identify the primary causes of mitochondrial dysfunction. According to oxygen consumption analysis, we observed that GM decreased the activity of complexes I and II which may explain the diminution in state 3 rates, RCI, and uncoupled respiration (Figures 7 and 8). These effects induced by GM on complexes I and II were recently known [49]. The absence of complex III activity alterations is in agreement with the previous observation of absence of changes in ascorbate-TMPD-supported respiration in isolated mitochondria exposed to GM [46]. Notwithstanding, complex IV activity was altered by GM exposure countering the statement that the terminal components of respiratory chain are relatively insensitive to GM effect [46]. Indeed, it has been demonstrated that cytochrome oxidase (complex IV) concentration and its activity decreased significantly after GM treatment in rats [49, 50]. Curcumin attenuating action in complex IV activity was consistent with its effects on state 3 and uncoupled respiration. Besides, several studies have shown the potential protective effect of curcumin on the respiratory chain complexes [10].

On the other hand, it is known that anion superoxide (O_2_^∙−^) generation is an important event in mitochondria exposed to GM* in vitro* [51]. Results in Figure 9(e) support this idea in our model as aconitase activity was strongly inhibited in GM group. Aconitase is an enzyme belonging to the tricarboxylic acids pathway and its activity can be used as a measure of mitochondrial oxidative stress [52]; specifically, aconitase activity inhibition can be used to indirectly determine O_2_^∙−^ production [32]. However, curcumin treatment produced a marginal nonsignificant recovery in aconitase activity probably because of the high sensitivity of this enzyme to GM-induced ROS production [25].

### 3.7. Ca^2+^-Dependent MPT

**Figure 10:**
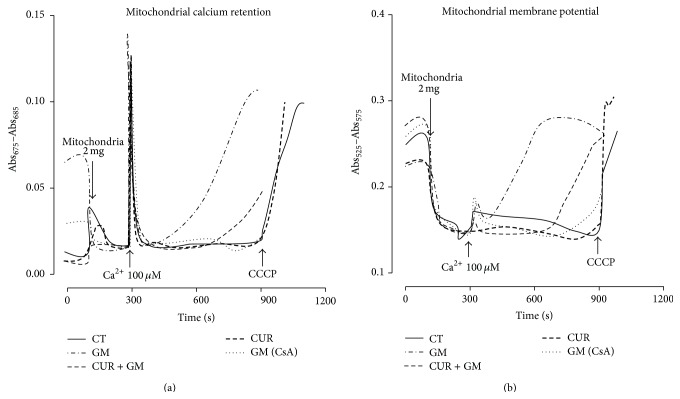
CUR treatment delays the Ca^2+^-dependent mitochondrial permeability transition in renal cortex of GM-exposed rats. (a) Representative tracings of Ca^2+^ retention capacity obtained by arsenazo III assay and (b) representative tracings of mitochondrial membrane potential responsive to Ca^2+^ overload obtained by safranin O assay. CCCP: carbonyl cyanide m-chlorophenylhydrazone; CT: control; GM: gentamicin; CUR: curcumin; CsA: cyclosporine A.

The effects of Ca^2+^ overload on the opening of the MPT pore evaluated as Ca^2+^ retention mitochondrial capacity and mitochondrial membrane potential are shown in Figures 10(a) and 10(b), respectively. Calcium was rapidly accumulated and maintained in mitochondria in all the experimental groups; however, high calcium concentration promoted the opening of the MPT pore in GM mitochondria, as CsA totally prevents this condition (Figure 10(a)). CUR partially mitigated Ca^2+^ release induced by GM. Ca^2+^ overload promotes membrane permeabilization and abolition of the mitochondrial membrane potential. In Figure 10(b), tracing GM evidences membrane potential dissipation next to Ca^2+^ addition. Unlike GM, CT and CUR tracings maintained their potential 600 s after Ca^2+^ addition, until CCCP was added. In CUR + GM mitochondria, depolarization was delayed as compared with GM mitochondria. The absence of this pattern in GM mitochondria with CsA confirms that potential loss is mediated by the formation of the MPT pore.

Ca^2+^ retention mitochondrial capacity and the membrane potential disruption by Ca^2+^ overload could be associated with the phenomenon of MPT as a triggering mechanism of cell death [53]. MPT pore opening is induced under pseudopathological conditions of oxidative stress. ROS production sensitizes mitochondria toward the MPT induction that, in turn, increases Ca^2+^ release which may enhance ROS production [54–56]. Thus, our results confirmed that mitochondria from GM-treated rats presented MPT pore opening, as previously described [57, 58]. CUR treatment favorably ameliorates the MPT pore opening from GM-treated rats protecting them from the noxious effects generated from this antibiotic by preserving mitochondrial integrity.

## 4. Conclusions

The main finding of this work was that the attenuation of GM-induced nephrotoxicity by curcumin was associated with an improvement of mitochondrial dysfunction. This improvement could be probably linked to a maintenance in the program of gene expression for mitochondrial components involved in respiratory chain that could result minimized by GM. Curcumin treatment attenuated the GM-induced alterations in mitochondrial energy-linked functions of renal cortex of rats, which are associated with protection of mitochondrial chain complexes and preserving mitochondrial integrity. Despite the well-known antioxidant properties of curcumin that can protect mitochondrial proteins against oxidative stress, another presumptive protective mechanism could be the preservation of mitochondrial biogenesis which would guarantee an opportune replacement of impaired structures. Mitochondrial biogenesis could be diminished by GM and curcumin would prevent this effect with participation of Nrf2 in some extension, another proposed mechanism of protection against GM-induced nephrotoxicity.
